# Ensemble streamflow forecasting based on variational mode decomposition and long short term memory

**DOI:** 10.1038/s41598-021-03725-7

**Published:** 2022-01-11

**Authors:** Xiaomei Sun, Haiou Zhang, Jian Wang, Chendi Shi, Dongwen Hua, Juan Li

**Affiliations:** 1 Shaanxi Provincial Land Engineering Construction Group Co., Ltd., Xi’an, 710075 China; 2 Institute of Land Engineering and Technology, Shaanxi Provincial Land Engineering Construction Group Co., Ltd., Xi’an, 710021 China; 3 Shaanxi Provincial Land Consolidation Engineering Technology Research Center, Xi’an, 710075 China; 4 Key Laboratory of Degraded and Unused Land Consolidation Engineering, Ministry of Natural Resources, Xi’an, 710075 China

**Keywords:** Hydrology, Hydrology

## Abstract

Reliable and accurate streamflow forecasting plays a vital role in the optimal management of water resources. To improve the stability and accuracy of streamflow forecasting, a hybrid decomposition-ensemble model named VMD-LSTM-GBRT, which is sensitive to sampling, noise and long historical changes of streamflow, was established. The variational mode decomposition (VMD) algorithm was first applied to extract features, which were then learned by several long short-term memory (LSTM) networks. Simultaneously, an ensemble tree, a gradient boosting tree for regression (GBRT), was trained to model the relationships between the extracted features and the original streamflow. The outputs of these LSTMs were finally reconstructed by the GBRT model to obtain the forecasting streamflow results. A historical daily streamflow series (from 1/1/1997 to 31/12/2014) for Yangxian station, Han River, China, was investigated by the proposed model. VMD-LSTM-GBRT was compared with respect to three aspects: (1) feature extraction algorithm; ensemble empirical mode decomposition (EEMD) was used. (2) Feature learning techniques; deep neural networks (DNNs) and support vector machines for regression (SVRs) were exploited. (3) Ensemble strategy; the summation strategy was used. The results indicate that the VMD-LSTM-GBRT model overwhelms all other peer models in terms of the root mean square error (RMSE = 36.3692), determination coefficient (R^2^ = 0.9890), mean absolute error (MAE = 9.5246) and peak percentage threshold statistics (PPTS(5) = 0.0391%). The addressed approach based on the memory of long historical changes with deep feature representations had good stability and high prediction precision.

## Introduction

Streamflow forecasting is of great significance for the optimal management and effective operation of a water resources system. Therefore, it has been investigated by many researchers, and numerous forecasting models have been developed in the past decades. Among these models, forecasting techniques based on statistical modeling, data-driven models, seem to be in fashion for their simplicity and robustness^1–3^. Regularization has played an important role in forecasting^4–6^.

These data-driven models can be classified into time series models and artificial intelligence (AI) models. Many previous researchers have applied time-series models to forecast streamflow, including autoregression (AR), moving average (MA), autoregressive moving average (ARMA), and autoregressive integrated moving average (ARIMA) models^7–9^. However, due to the linear hypothesis of these models, they are not suited to forecast streamflow with non-linear and non-stationary characteristics. Therefore, AI models that have the ability of non-linear mapping are applied to streamflow forecasting, i.e., support vector machines for regression (SVRs)^1,10^, fuzzy inference systems (FIS)^11,12^, Bayesian regression (BR)^13,14^, and artificial neural networks (ANNs)^15,16^. However, most of the AI models, which belong to the “shallow” learning category, cannot sufficiently represent instinctual information^17^. The deep learning models, e.g., the deep belief network (DBN) and recurrent neural networks (RNNs), can overcome this drawback due to their deeper representation ability^17^. However, these deep learning models completely rely on historical observed data, and some of the earlier changes of streamflow may or may not influence future streamflow. It is entirely possible for the gap between the streamflow information from further back in time and the current point where it is needed to become large. Therefore, using a deep learning model that can automatically “remember” or “forget” previous information should be able to enhance the accuracy of streamflow forecasting. Fortunately, LSTM^18^, one of the deep learning models, has the ability to tackle this task. LSTM has been successfully used in some fields, e.g., accident diagnosis^19^, electricity price prediction^20^, water table depth forecasting^21^, and others.

Unfortunately, due to the complicated non-linearity, extreme irregularity and multiscale variability of natural streamflow, the models directly built on original streamflow cannot appropriately identify streamflow change patterns^1^. For this reason, the processes of transformation, data pre-processing and feature extraction have attracted the attention of many researchers. In addition, feature extraction can efficiently improve the capability of these models^1,17,22,23^. Huang et al.^1^ applied a modified empirical model decomposition (EMD) method to remove the most nonlinear and disorderly noise from the original series and then established one SVR-based model that computed a summation of all prediction results of all sub-series as an ensemble result. Wang et al.^24^ used the EEMD technique to develop insight into the runoff characteristics and forecast each characteristic by the ARIMA model; the forecast results were summed to formulate an ensemble forecast for the raw runoff. Bai et al.^17^ used EEMD to extract multi-scale features and reconstructed three deep neural networks (DNNs) by a summing strategy to forecast reservoir inflow. Yu et al.^10^ exploited both Fourier transform (FT) and SVR models to extract and learn features and to forecast monthly reservoir inflow by adding all feature learning results. Obviously, feature representation of original data can contribute to the performance improvement of streamflow forecasting because of the advantage of feature extraction, which removes noise components and detect the hidden structures inside the raw time-series.

However, some recent commonly-used feature extraction methods, e.g., EMD, EEMD, and wavelet transforms (WT), suffer from drawbacks with respect to different aspects of actual signal decomposition. For example, EMD has limitations of sensitivity to noise and sampling^25^, EEMD is not theoretically well-founded^26^, and the effectiveness of WT heavily relies on the choice of the basic wavelet function^27^. Recently, a theoretically well-founded and robust VMD model^25^ has been successfully applied to container throughput forecasting^28^, vibro-acoustic feature extraction^29^, chatter detection in milling processors^30^, and other applications. This model is much more sensitive to noise and sampling than existing decomposition algorithms, such as EMD and EEMD^25^.

Moreover, the ensemble strategy plays a vital role for integrating feature forecasting results to predict original streamflow. A straightforward and frequently used ensemble technique is summation, although it may cause error accumulation problems due to summation errors of the sub-results of streamflow prediction. Sometimes, the gap between the summation of extracted features and the original values may not be small. Even a model that can achieve great performance in the prediction of sub-features may still not be able to accurately forecast the original time series. Therefore, building another supervised model^3^, such as GBRT, for ensembles seems to be a good choice to avoid an accumulation of errors and obtain better performance.

Based on the above outline, this study addresses a decomposition-ensemble-based multiscale feature deep learning method to forecast streamflow. Our goal is to plug a memory framework into the process of deep feature learning that is robust to noise and sampling as well as long historical changes of streamflow, integrate an ensemble tree model with the capability to remove impacts caused by error accumulation and model the relationship between decomposition results and the original series to exploit the sophisticated nature of streamflow with a long history. To this end, VMD was used to extract smooth features, LSTM was applied to learn features sensitive to long historical streamflow changes, and GBRT was used to obtain an ensemble model to forecast the streamflow. This approach was evaluated by observed daily streamflow of the Yangxian station, Han River, China.

## Methodologies

### Variational mode decomposition (VMD)

The VMD algorithm, an entirely non-recursive variational mode decomposition model proposed by Dragomiretskiy and Zosso^25^, is used to concurrently decompose a sophisticated signal into several band-limited intrinsic modes.

The VMD model assumes each mode ${u}_{k}$ to be mainly compact around a center pulsation $\omega_{k}$ calculated with the decomposition. The following scheme proposed by Dragomiretskiy and Zosso^25^ is applied to assess the bandwidth of a mode. The related analytic signal of each mode $u_{k}$ is first computed by the Hilbert transform to acquire a unilateral frequency spectrum. Then, the frequency spectrum of each model is shifted to “baseband” by mixing with an exponential tuned to the respective evaluated center frequency. The bandwidth is finally assessed by the ${H}^{1}$ Gaussian smoothness of the demodulated signal, i.e., the squared *L*^2^-norm of the gradient^25^. To solve the decomposition problem of time series $f$, the constrained variational problem can be equivalently solved by the following equation:

1 $$ \left\{ {\begin{array}{*{20}c} {\mathop {\min }\limits_{{\left\{ {u_{k} } \right\},\left\{ {\omega_{k} } \right\}}} \left\{ {\sum\nolimits_{k} {\left\| {\partial_{t} \left[ {\left( {\delta \left( t \right) + \frac{j}{\pi t}} \right) * u_{k} \left( t \right)} \right]e^{{ - j\omega_{k} t}} } \right\|_{2}^{2} } } \right\}} \\ {s.t.\quad \sum\nolimits_{k} {u_{k} \left( t \right) = f\left( t \right)} } \\ \end{array} } \right. $$

where $\left\{ {u_{k} } \right\}: = \left\{ {u_{1} , \ldots ,u_{k} } \right\}$ and $\left\{ {\omega_{k} } \right\}: = \left\{ {\omega_{1} , \ldots ,\omega_{k} } \right\}$ denote the set of modes and their center frequencies, respectively. To solve this variational problem, a Lagrangian multiplier $\lambda$ and a quadratic penalty term are introduced to render the problem unconstrained. The augmented Lagrangian $\ell$ is defined as follows:

2 $$ \begin{aligned} \ell \left( {\left\{ {u_{k} } \right\},\left\{ {\omega_{k} } \right\},\lambda } \right) & : = \alpha \sum\limits_{k} {\left\| {\partial_{t} \left[ {\left( {\delta \left( t \right) + \frac{j}{\pi t}} \right) * u_{k} \left( t \right)} \right]e^{{ - j\omega_{k} t}} } \right\|_{2}^{2} } + \left\| {f\left( t \right) - \sum\limits_{k} {u_{k} \left( t \right)} } \right\|_{2}^{2} \\ & \quad + \left\langle {\lambda \left( t \right),f\left( t \right) - \sum\limits_{k} {u_{k} \left( t \right)} } \right\rangle \\ \end{aligned} $$

in which $\alpha$ indicates the balancing parameter of the data-fidelity constraint. In VMD, the Alternate Direction Method of Multipliers (ANMM) is used to solve Eq. (2). Equation (3) is used to update the mode $u_{k} \left( \omega \right)$ in the frequency domain. The center frequencies $\omega_{k}$ are updated by Eq. (4), and $\lambda$ is simultaneously updated by Eq. (5). In the time domain, the mode $u_{k} \left( t \right)$ can be obtained as the real part of the inverse Fourier transform of $u_{k} \left( \omega \right)$ expressed by Eq. (3)

3 $$ \hat{u}_{k}^{n + 1} \left( \omega \right) = \frac{{\hat{f}\left( \omega \right) - \sum\limits_{i \ne k} {\hat{u}_{i} } + \frac{{\hat{\lambda }\left( w \right)}}{2}}}{{1 + 2\alpha \left( {\omega - \omega_{k} } \right)^{2} }} $$

4 $$ \hat{\omega }_{k}^{n + 1} = \frac{{\int_{0}^{ \propto } {\omega \left| {\hat{u}_{k} \left( \omega \right)} \right|^{2} d\omega } }}{{\int_{0}^{ \propto } {\left| {\hat{u}_{k} \left( \omega \right)} \right|^{2} d\omega } }} $$

5 $$ \hat{\lambda }^{n + 1} \left( \omega \right) = \hat{\lambda }^{n} \left( \omega \right) + \tau \left( {\hat{f}\left( \omega \right) - \sum\limits_{k} {\hat{u}_{k}^{n + 1} \left( \omega \right)} } \right) $$

The implementation process of the VMD model is summarized as Algorithm 1.
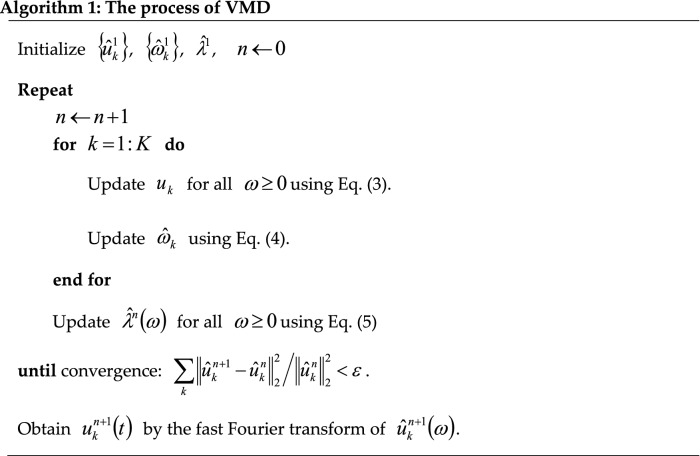


### Long short-term memory (LSTM)

**Figure 1:**
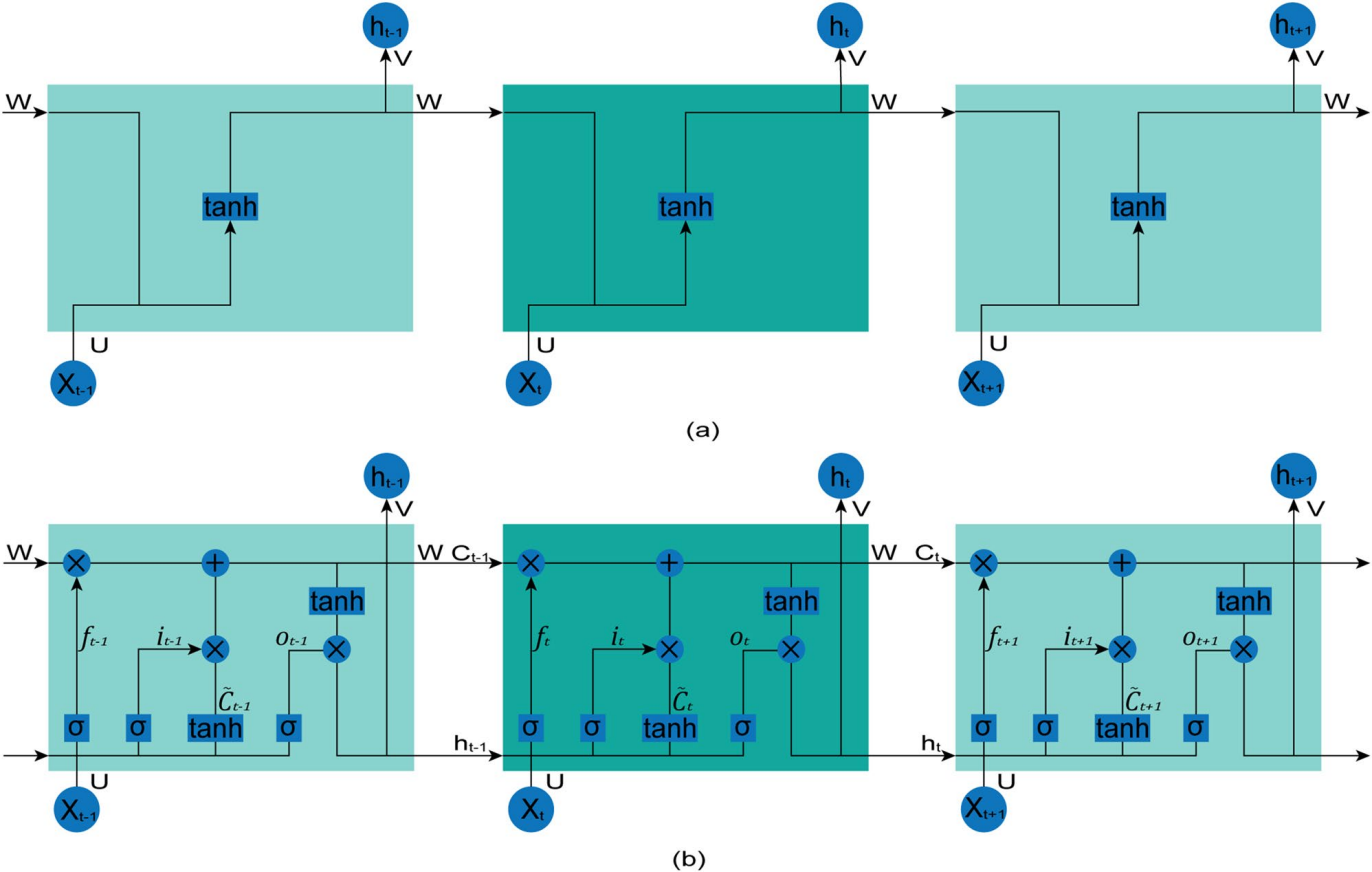
(**a**) Chain-like structure of the RNN. Because of the connections between hidden units, information can be passed from one time step to the next. (**b**) A graphical representation of the LSTM recurrent network with the memory cell block.

Long short-term memory networks (LSTMs) are a very specific kind of Recurrent Neural Networks (RNNs) for modeling sequential data. Therefore, it is essential to first introduce a normal version of an RNN. RNNs have chain-like structures of repeating modules that produce an output at each time step and have recurrent connections from the output at one time step to the hidden units at the next time step, illustrated in Fig. 1a. The chain-like structure with self-connected hidden units can help RNNs to “remember” the previous information, which allows the RNNs to build a model for an arbitrarily-long time sequence.

The forward propagation algorithm is used to calculate the output for the RNN pictured in Fig. 1a. Begin with a specification of the initial state $h_{0} = 0$ for each time step from $t = 1$ to $t = \tau$; the following equations are used^31^:

6 $$ h_{t} = \tanh \left( {Wh_{t - 1} + UX_{t} + b_{h} } \right) $$

7 $$ o_{t} = Vh_{t} + b_{o} $$

where the parameters are the bias vectors ${\mathrm{b}}_{\mathrm{h}}$ and ${\mathrm{b}}_{\mathrm{o}}$ along with the weight matrices *U*, *V* and *W* for input-to-hidden, hidden-to-output and hidden-to-hidden connections, respectively. ${\mathrm{X}}_{\mathrm{t}}$ represents the input vector at time step $\mathrm{t}$ and $h_{t - 1}$ denotes the hidden cell state at time step $t-1$.

Back-Propagation Through Time (BPTT) is used to compute the gradients of the RNNs^32^. However, owing to the gradient vanishing or exploding problem, it is difficult and inefficient for BPTT to learn long-term dependencies in RNNs^33,34^. LSTMs are explicitly designed by Hochreiter and Schmidhuber^18^ to avoid this long-term dependency problem. LSTMs also have chain-like repeating modules, although with complicated structures. Each repeating module of LSTMs includes a memory block called a “cell”. This memory block help LSTMs to store or remove information over a long duration.

The LSTM memory block diagram is illustrated in Fig. 1b. The LSTM memory block contains four parts, a cell state in addition to three special structures called gates. The horizontal line running through the top of the diagram is the cell state, which runs straight down the entire chain without any activation function; it is very easy for information to just flow along it unchanged. Therefore, the gradient does not vanish or explode when training an LSTM by BPTT. Moreover, the LSTM does have the ability to add or remove information to the cell state, regulated by the input, forget and output gates. Each gate is composed of a sigmoid unit and a pointwise multiplication operation, which can optionally pass information.

The corresponding forward propagation equations of LSTM are expressed for time steps from $t = 1$ to $t = \tau$ with initial state $C_{0} = 0$ and $H_{0} = 0$ as:

8 $$ i_{t} = \sigma \left( {W_{i} X_{t} + U_{i} h_{t - 1} + b_{i} } \right) $$

9 $$ f_{t} = \sigma \left( {W_{f} X_{t} + U_{f} h_{t - 1} + b_{f} } \right) $$

10 $$ o_{t} = \sigma \left( {W_{o} X_{t} + U_{o} h_{t - 1} + b_{o} } \right) $$

11 $$ \tilde{C}_{t} = \tanh \left( {W_{C} X_{t} + U_{C} h_{t - 1} + b_{C} } \right) $$

12 $$ C_{t} = f_{t} \otimes C_{t - 1} + i_{t} \otimes \tilde{C}_{t} $$

13 $$ h_{t} = o_{t} * \tanh \left( {C_{t} } \right) $$

where *W*, *U* and *b* are input weights, recurrent weights and biases, respectively, and the subscripts $i$, $f$ and $o$ represent the input, forget and output gates, respectively. The activation function *logistic sigmoid* is indicated by $\sigma$. $i_{t}$, $f_{t}$, $o_{t}$ and $C_{t}$ are the input, forget, output gates and cell state vectors at time step $t$, respectively. $h_{t}$ is the output vector of the memory cell block, and $\otimes$ denotes element-wise multiplication.

### Gradient Boosting Regression Trees (GBRTs)

Gradient boosting is a powerful machine learning strategy to efficiently produce highly robust, competitive, interpretable procedures for both regression and classification^35,36^. The key to boosting is to combine the output of many weak prediction models (“learners”), typically decision trees, into a single strong ensemble model. Gradient boosting builds models in a forward stage-wise fashion. Therefore, for each stage $m$, $1 \le m \le M$, of gradient boosting:

14 $$ F_{m} \left( x \right) = F_{m - 1} \left( x \right) + h_{m} \left( x \right) $$

in which $h_{m} \left( x \right)$ are the basic estimators referred to as weak prediction models (small regression trees in the case of GBRT) and $F_{m} \left( x \right)$ is the summation of $m$ small regression trees for GBRT. For iterations from $m = 1$ to $m = M$, the GBRT algorithm improves on $F_{m}$ by adding a new regression tree $h_{m}$ to its predecessor to provide a better model. Simultaneously, the procedure estimates the target value $y$ based on the perfect $h_{m}$ from the training set, which would imply:

15 $$ F_{m} \left( x \right) = F_{m - 1} \left( x \right) + h_{m} \left( x \right) = y $$

which is equivalent to

16 $$ h_{m} \left( x \right) = y - F_{m - 1} \left( x \right) $$

Therefore, $h_{m}$ is the regression tree model that fits the current residuals $\gamma_{m} = y - F_{m - 1} \left( x \right)$, and the residuals $y - F_{m - 1} \left( x \right)$ for a given model are the negative of the squared error loss function, i.e.:

17 $$ - \frac{{\partial \frac{1}{2}\left( {y - F_{m - 1} \left( x \right)} \right)^{2} }}{{\partial F_{m - 1} \left( x \right)}} = y - F_{m - 1} \left( x \right) $$

Gradient boosting is thus a gradient descent algorithm obviously proved by Eq. (17), and generalizing it entails substituting the squared error with a different loss function and its gradient. For a more detailed description, see Friedman^35^ and Hastie et al.^37^.

Moreover, the implicit idea behind gradient boosting is to apply a steepest-descent step to minimize the loss values between the response values and estimates to find an optimal approximation $\hat{F}\left( x \right)$. Therefore, for a training set $\left\{ {\left( {x_{1} ,y_{1} } \right), \ldots ,\left( {x_{n} ,y_{n} } \right)} \right\}$, the ensemble model would be updated in accordance with the following equations^35,37^:

18 $$ F_{m} \left( x \right) = F_{m - 1} \left( x \right) - \gamma_{m} \sum\limits_{i = 1}^{n} {\frac{{\partial L\left( {y_{i} - F_{m - 1} \left( {x_{i} } \right)} \right)}}{{\partial F_{m - 1} \left( {x_{i} } \right)}}} $$

19 $$ \gamma_{m} = \arg \mathop {\min }\limits_{\gamma } \sum\limits_{i = 1}^{n} {L\left( {y_{i} - F_{m - 1} \left( {x_{i} } \right) - \gamma \frac{{\partial L\left( {y_{i} ,F_{m - 1} \left( {x_{i} } \right)} \right)}}{{\partial F_{m - 1} \left( {x_{i} } \right)}}} \right)} $$

where the derivatives are obtained with respect to the function $F_{i}$ for $i \in \left\{ {1,2, \ldots ,m} \right\}$. In the *m*-th iteration of the GBRT model, the gradient boosting algorithm fits a regression tree $h_{m} \left( x \right)$ to the pseudo-residuals. Let $J_{m}$ be the number of tree leaves; the regression tree splits the input space into $J_{m}$ disjoint regions $R_{1m} , \ldots ,R_{{J_{m} m}}$ and obtains a constant value for each region. The output of $h_{m} \left( x \right)$ for input $x$ can thus be written as the sum^35^:

20 $$ h_{m} \left( x \right) = \sum\limits_{j = 1}^{{J_{m} }} {b_{jm} } 1_{{R_{jm} }} \left( x \right),\left( {x \in R_{jm} } \right) $$

where $b_{jm}$ is the constant value predicted for the region $R_{jm}$ and $1\left( \cdot \right)$ is an indicator function that has the value 1 if its argument is true and zero otherwise. Then, each coefficient $b_{jm}$ is multiplied by an optimal value $\gamma_{jm}$^35^, and the model is then updated by the following rules:

21 $$ F_{m} \left( x \right) = F_{m - 1} \left( x \right) + \sum\limits_{j = 1}^{{J_{m} }} {\gamma_{jm} 1_{{R_{jm} }} \left( x \right),\left( {x \in R_{jm} } \right)} $$

22 $$ \gamma_{jm} = \arg \mathop {\min }\limits_{\gamma } \sum\limits_{i = 1}^{n} {L\left( {y_{i} ,F_{m - 1} \left( {x_{i} } \right) + \gamma } \right)} $$

The implementation process of the generic gradient boosting tree is summarized as Algorithm 2.
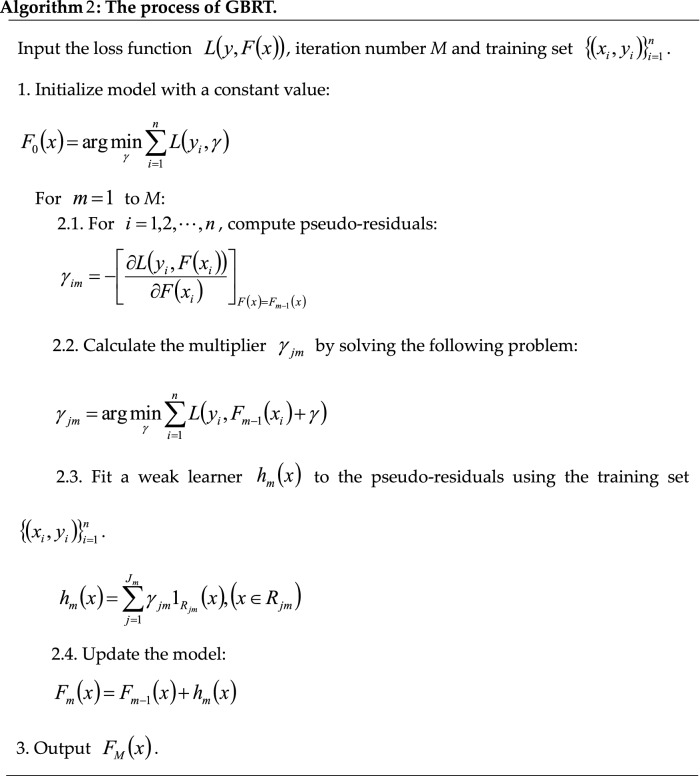


### The decomposition-ensemble model VMD-LSTM-GBRT

**Figure 2:**
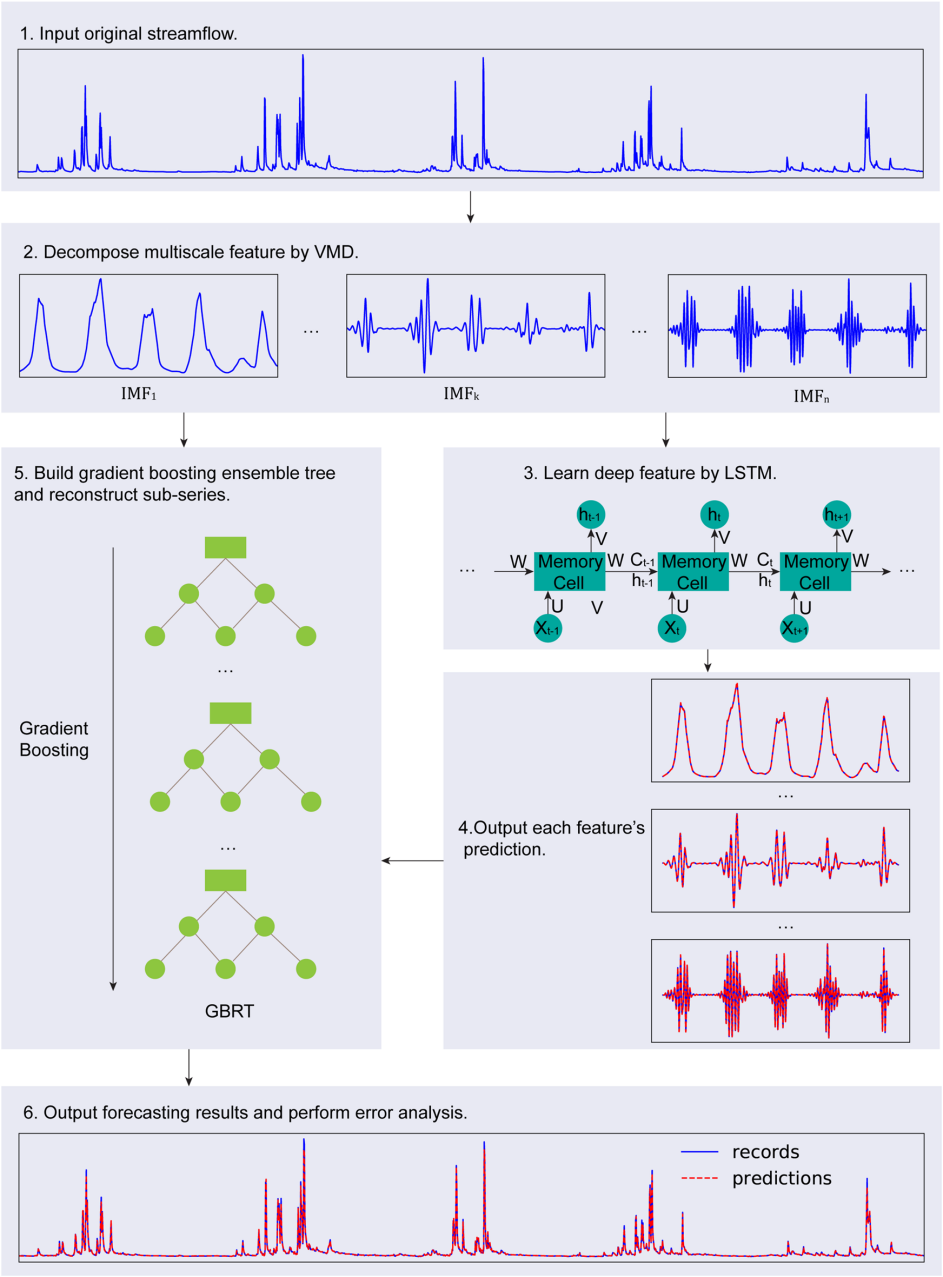
Application of the proposed model VMD-LSTM-GBRT.

After discussing each key constituent separately, the approach of the proposed model VMD-LSTM-GBRT can be concluded as follows and is diagrammed in Fig. 2.

**Step 1**. Collect raw daily streamflow data $X = \left\{ {x_{1} ,x_{2} , \ldots ,x_{N} } \right\}$.

**Step 2**. Use VMD to decompose the raw series $X$ into several components.

**Step 3**. Plot the partial autocorrelation coefficient figure of each component obtained in step 2 to select optimal numbers of inputs for it. Divide each of the components into three sub-sets: the training set (80%) for training multiple LSTM structures, the development set (10%) for searching optimal structure, and the test set (10%) for validating the ensemble model VMD-LSTM-GBRT.

**Step 4**. Given the test set, predict each component based on the optimal LSTM structure of each mode obtained in step 3.

**Step 5**. Build the ensemble tree model GBRT using the components obtained in step 2 as input and the original series obtained in step 1 as output. Use GBRT to reconstruct the predictions given by step 4.

**Step 6**. Output the forecasting streamflow results and perform error analysis.

## Case study

### Study site and observational data

**Figure 3:**
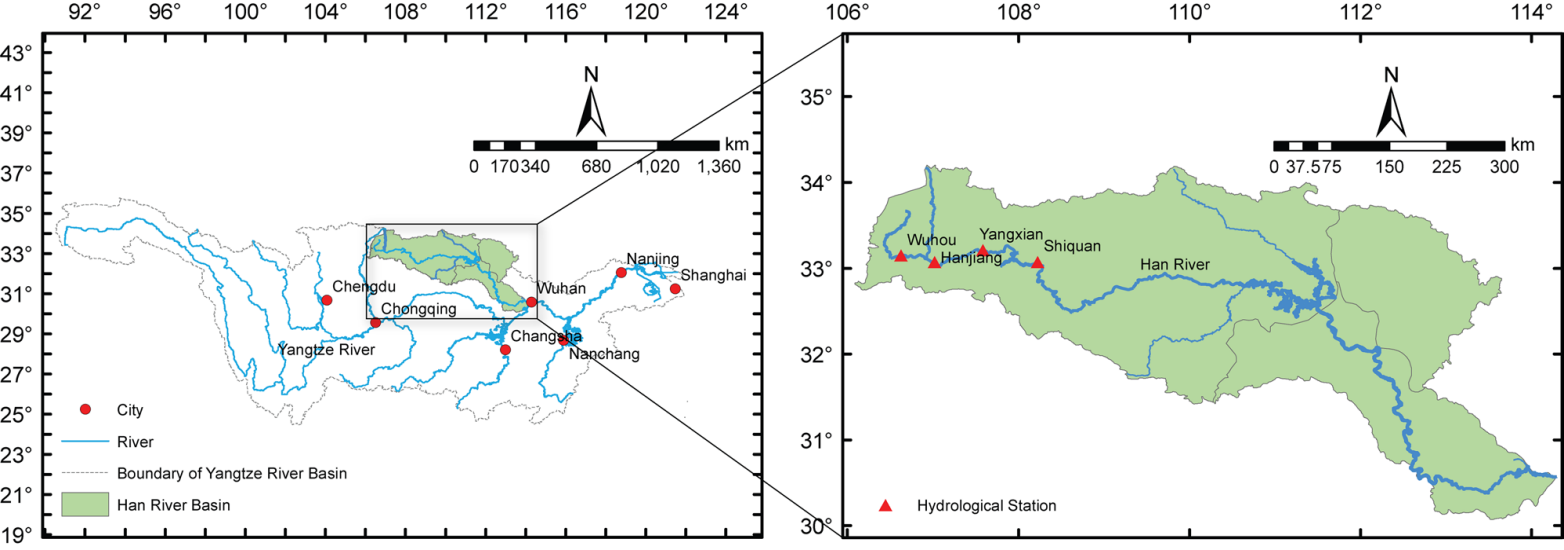
Location of the Yangxian hydrological station.

In this paper, historical daily streamflow of the Yangxian hydrological station on the upstream of Han River were collected to assess the proposed model. The location of Yangxian station is illustrated in Fig. 3. The Han River, the biggest tributary of the Yangtze River, lies within 30°–34.5° N and 107°–114.5° E in the middle of China and has a total drainage area of 151,000 km^2^. The location of this basin is in a subtropical monsoon zone that has a humid climate and differentiated seasons. The water resources in this area are rich, with an annual average precipitation of more than 900 mm/year. The precipitation in the rainy season (July to September) accounts for 75% of the annual total, and the runoff has a similar seasonality. The study area of this paper is the upper source area of the Han River, which lies between BoZhong Mountain located in the south of the Qinling Mountains and the Yangxian hydrological station. The drainage area controlled by the Yangxian hydrological stations is 14,484 km^2^. As the main stream control station, forecasting the daily runoff of the Han River at Yangxian hydrological station can evaluate the short-term water production at the source of this basin.

**Figure 4:**
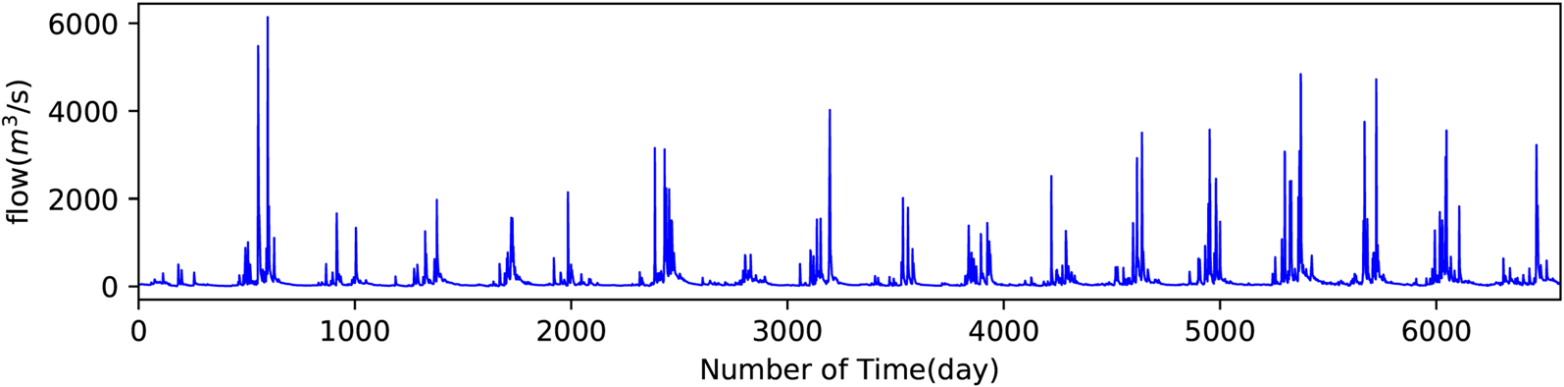
Daily Streamflow of the Yangxian station from 1/1/1997 to 31/12/2014.

As shown in Fig. 4, daily streamflow records (total of 6574 samples) of the Yangxian hydrological station from 1/1/1997 to 31/12/2014 were used to develop the present model. For simplicity, the observation dates on the horizontal axis have been replaced with series numbers. These records were collected from the hydrological information datacenter of Shaanxi Hydrographic and the Water Resources Survey Bureau. The instantaneous value (m^3^/s) observed at 8 a.m. was selected as the average daily streamflow.

### Data pre-processing

Since the range of the streamflow time series and its decomposition sequences vary widely, in some cases of building machine learning models, the optimization algorithms applied for the loss function will not work well without feature normalization. Therefore, all the variables used for the model developed in the present study were normalized to the same scale. This pre-processing strategy can ensure that the optimization algorithm converges much faster than without normalization^38^. The normalization formula is as follows:

23 $$ X_{normalized} = 2 * \frac{{X - X_{\min } }}{{X_{\max } - X_{\min } }} - 1 $$

where $X_{normalized}$ is the normalized vector and $X$ is the raw vector. $X_{\max }$ and $X_{\min }$ are the maximum value and minimum value of $X$, respectively, and $X_{normalized}$ is calculated by element-wise mathematical operations. Once we have finished the simulation, the predictions can be re-normalized following the inverse procedure of Eq. (23).

### Model evaluation criteria

The hidden layer is set with 1, 2, 3, 4 and 5; The learning rate is set to 0.001, 0.003, 0.007, 0.01, 0.03, 0.07, 0.1; The number of hidden layer neurons is set to 1–25, and the other parameters use the default parameters used by TensorFlow (the activation function of each layer is Rectified Linear Unit, the optimization algorithm is Adam algorithm, the loss function is mean squared error, the kernel initializer is Xavier uniform initialization, the bias initializer is zero initialization).

To evaluate the performance of the proposed model based on the decomposition-ensemble strategy, four error analysis criteria were applied. The expression of these criteria is shown in Table 1. The RMSE evaluates the performance of predicting high streamflow values, whereas the MAE accesses the average performance of the entire data set. The coefficient of determination $R^{2}$ indicates how well the observations are replicated by the proposed model. The peak percentage of threshold statistics, PPTS, denotes the ability to forecast peak flow^17,39^. The lower the PPTS, the better the capability to forecast peak flow. Note that the records are arranged in descending order to compute the PPTS and that the threshold level γ denotes the percentage of bottom data removed from this order; the parameter G is the number of top data at the threshold level γ.

**Table 1:** Formulas for error analysis criteria.

Error analysis criteria	Definition
Root mean square error	$RMSE = \sqrt {\frac{{\sum\nolimits_{t = 1}^{N} {\left( {x\left( t \right) - \hat{x}\left( t \right)} \right)^{2} } }}{N}}$
Mean absolute error	$MAE = \frac{{\sum\nolimits_{t = 1}^{N} {\left| {x\left( t \right) - \hat{x}\left( t \right)} \right|} }}{N}$
Determination coefficient	$R^{2} = 1 - \frac{{\sum\nolimits_{t = 1}^{N} {\left( {x\left( t \right) - \hat{x}\left( t \right)} \right)^{2} } }}{{\sum\nolimits_{t = 1}^{N} {\left( {x\left( t \right) - \overline{x}\left( t \right)} \right)^{2} } }}$
Peak percentage threshold statistics (%)	$PPTS\left( \gamma \right) = \frac{1}{100 - \gamma }\frac{1}{N}\sum\limits_{t = 1}^{G} {\left| {\frac{{x\left( t \right) - \hat{x}\left( t \right)}}{x\left( t \right)} \cdot 100} \right|}$

*N* is the number of samples, $x\left( t \right)$ is the original series, $\overline{x}\left( t \right)$ is the average of the original series and $\hat{x}\left( t \right)$ is the predicted series.

## Results and discussion

### Data decomposition with VMD

**Figure 5:**
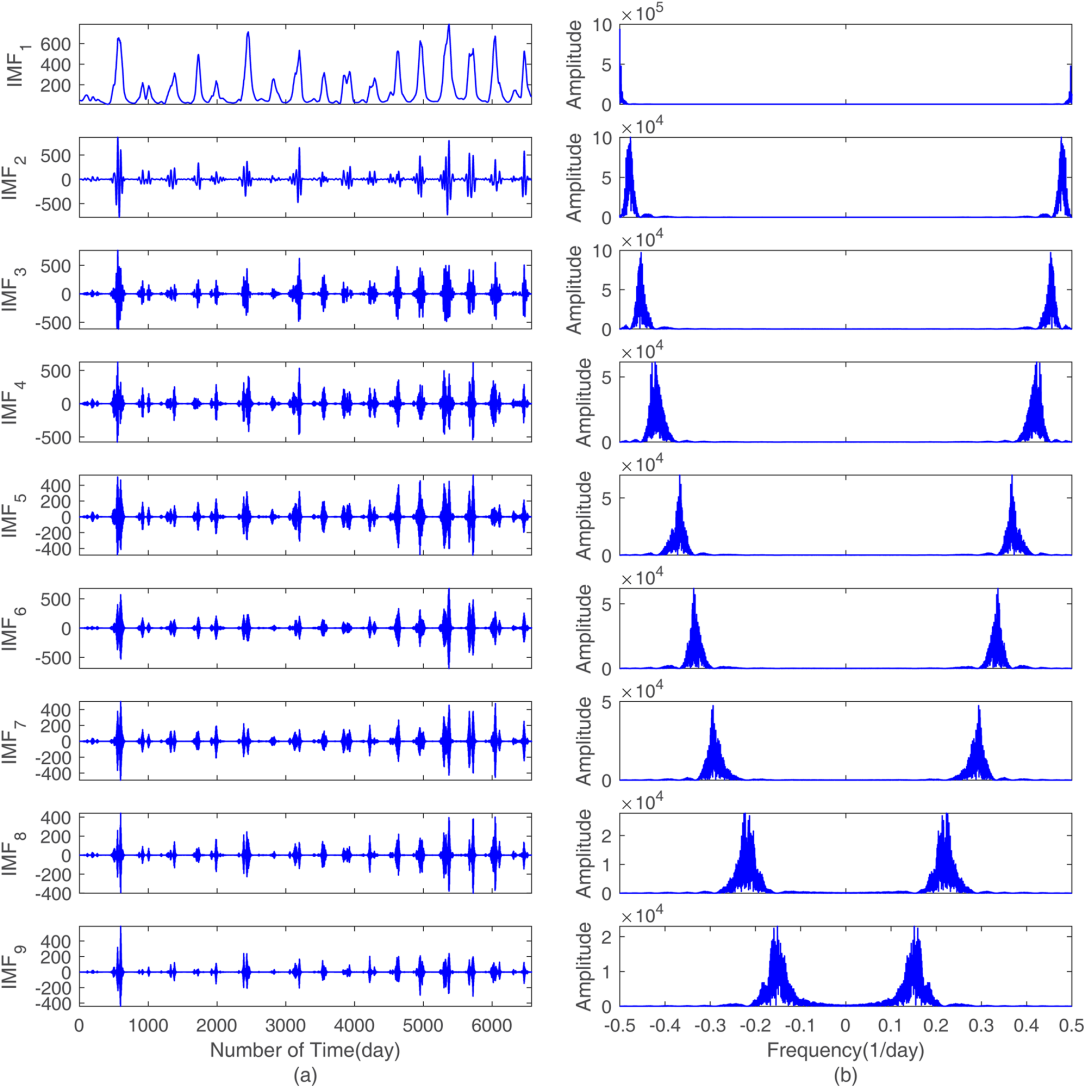
VMD decomposition results: (**a**) the decomposition sequence waveform and (**b**) the frequency spectrum representation.

**Figure 6:**
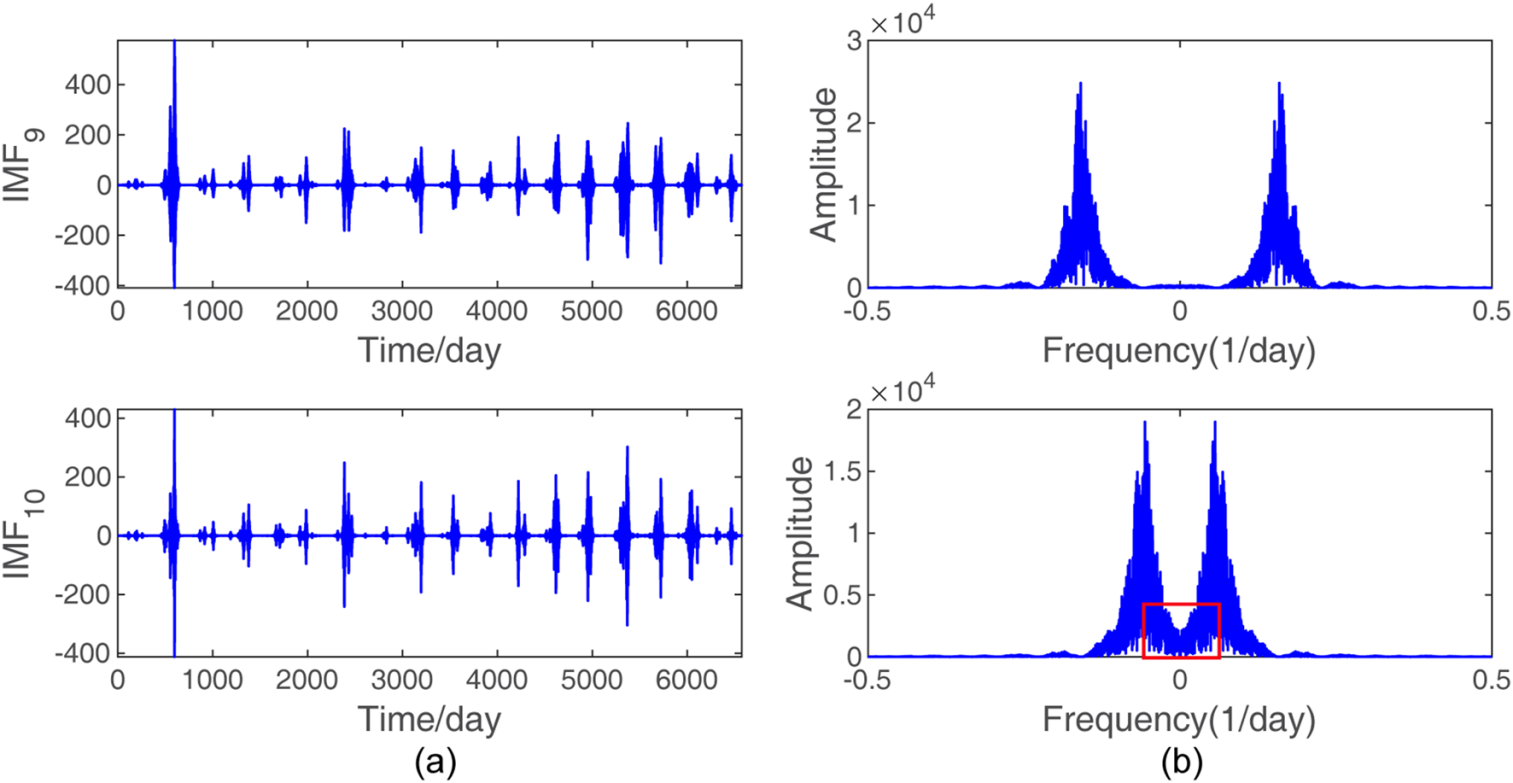
Schematic diagram of the center frequency aliasing of the last IMF: (**a**) the last two sequence waveforms and (**b**) the frequency spectrum representations. The area surrounded by the red rectangular border indicates the aliasing.

As mentioned in “The decomposition-ensemble model VMD-LSTM-GBRT”, when building the decomposition-ensemble based model, we first decomposed the raw daily streamflow data of the Yangxian hydrological station via VMD. The raw series and its decomposition results and the frequency spectra are shown in Fig. 5. However, it is hard to tell how many components the original series should be decomposed into. Too few components may not properly extract features inside raw data, whereas too many may be computationally expensive for training the model. By experiments, we found that the optimal decomposition mode number can be determined by the obvious aliasing phenomenon of the center frequency for the last component. It was first found in this study that when *k* = 10, the frequency spectrum of the 10th mode had obvious aliasing phenomena (area surrounded by a red rectangular border shown in Fig. 6). To make the decomposition result satisfy orthogonality and avoid the spurious components as much as possible, the number of components was chosen to be 9.

### Multiscale deep feature learning with LSTM

**Figure 7:**
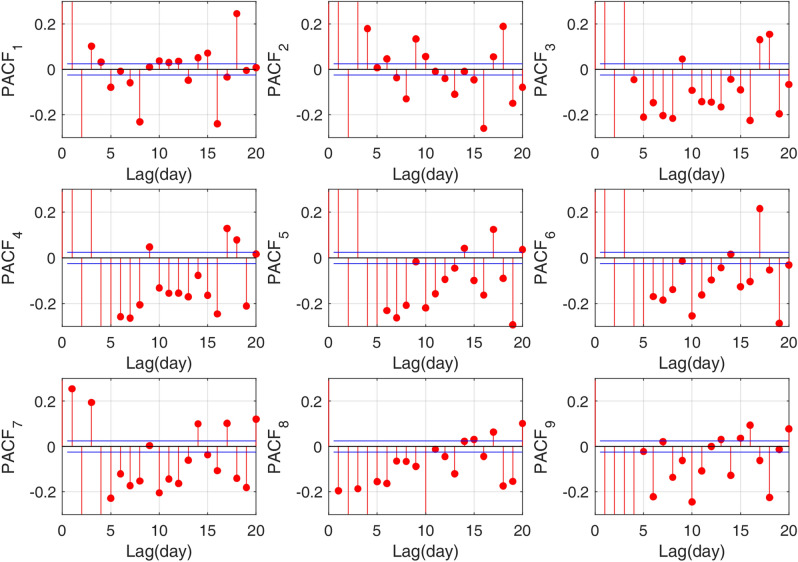
PACFs of subseries of daily streamflow during the period 1997/01/01 to 2014/12/31 for the Yangxian hydrological station.

The numbers of inputs, network structure and other hyper-parameters are vital variables in an LSTM model. Therefore, according to the input selection method introduced by He et al.^11^, Huang et al.^1^, and Wang et al.^16^, the input variables could first be easily obtained by observing the plot of Partial Autocorrelation Functions (PACFs) illustrated by Fig. 7, in which PACF_1_-PACF_9_ denotes the PACFs of each component. In other words, we assume that the output is $X\left( {t + 1} \right)$ and $X\left( {t + 1 - k} \right)$ is then selected as one of the input variables under the condition that PACF at lag *k* is out of the 95% confidence interval indicated by the blue lines in Fig. 7. Figure 7 shows that almost all the PACFs of each component are out of the range. Therefore, we select the 20 days of lag form $X\left( {t - 19} \right)$ to $X\left( t \right)$ as the input variables of each sub-series.

Since there is no mature approach in theory to determine the number of hidden units and hidden layers, an experimental method can be used to select these two parameters for the LSTM network structure. In the experiment of this study, the sub-series shown in Fig. 5, first normalized by Eq. (23), were split into three parts: the training data set (1/1/1997–27/5/2011), the development set (28/5/2011–14/3/2013) and the testing set (15/3/2013–31/12/2014). The training set is used to train the model, that is, to train the parameters such as weight matrix and threshold in LSTM model; The development set is used to optimize model super parameters, such as learning rate, number of network neurons, number of network layers, etc.; The testing set is used to validate the accuracy of the model and to show the confidence level. The number of hidden units for each hidden layer was designed for 11 levels ranging from 15 to 25, and the number of input variables equaled the median of this interval. The hidden layers of LSTM were initialized from 1 to 5. Therefore, for each component of the original series, there were 55 LSTM network structures in this experiment. Training and developing the LSTM model to predict the first component, IMF_1_, is an example used to describe the experimental process.

**Figure 8:**
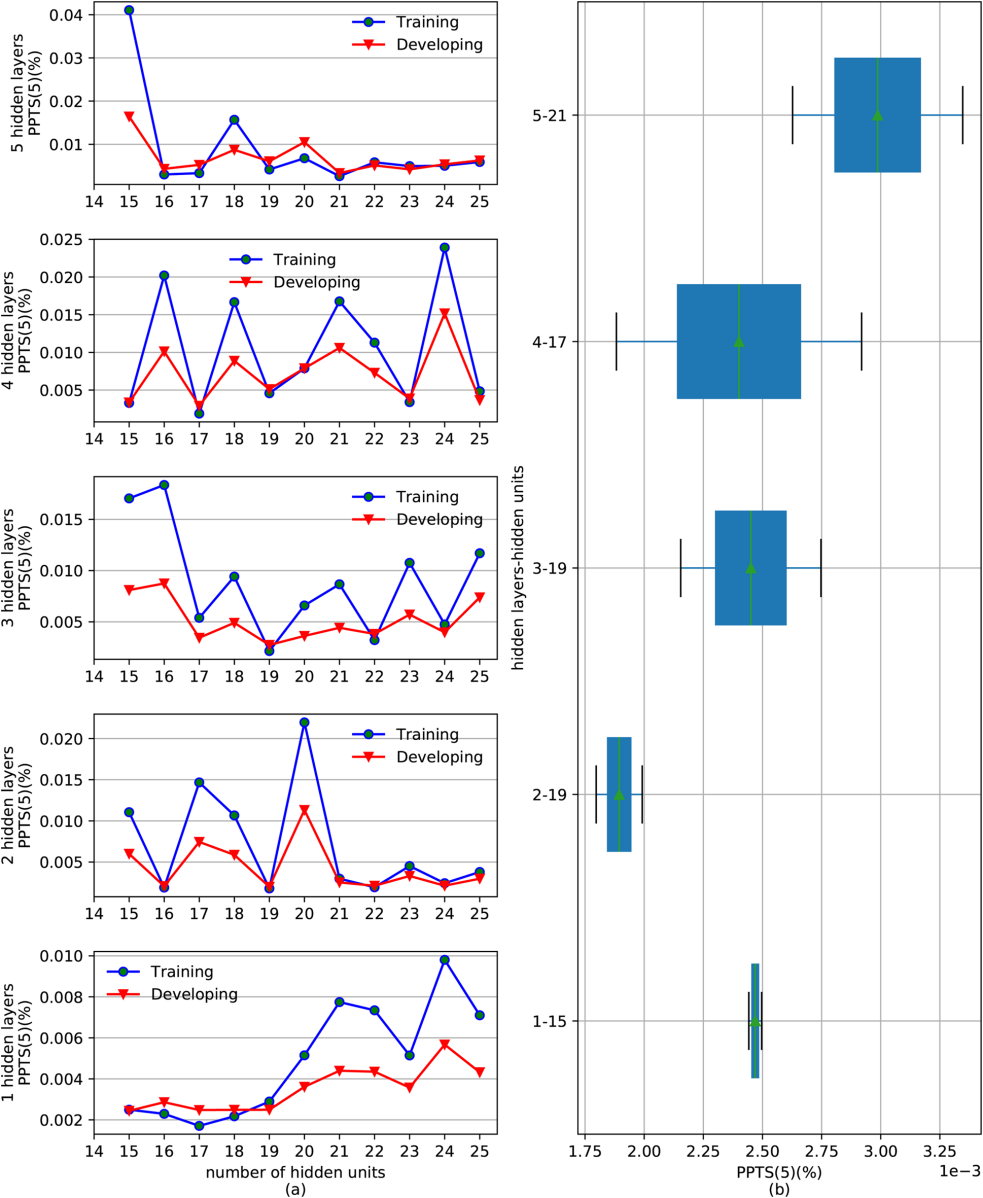
PPTS(5) of different LSTM structures for predicting streamflow of IMF_1_ during the training and development period: (**a**) line chart plots of PPTS(5) for different hidden layers and (**b**) boxplots of the optimal structure of each hidden layer.

For each network structure mentioned in the previous paragraph, we first initialized an LSTM model with 20 input units and then trained these 55 structures using the training set. Then, the streamflow during the development period was forecast based on the trained structures. To find the optimal model structure, PPTS(5), illustrated in Fig. 8, was calculated for the training and development set. Figure 8a shows the changes of PPTS(5) of the training and development set for different levels of the hidden layers. According to the rule of bias and variance tradeoff^40^, when the PPTS(5) of the development set is close to the PPTS(5) of the training set and both of these values are small, the model structure will obtain a great generalization and predicting ability. Therefore, we selected 20–15–1, 20–19–19–1, 20–19–19–19–1, 20–17–17–17–17–1 and 20–21–21–21–21–21–1 as the optimal structure of the 1–5 hidden layers, respectively. 20–19–19–1 means that the structure has 20 input features, 1 output target, and two hidden layers, with 19 hidden units for each layer. Figure 8b shows the boxplots of optimal structure for each level of the hidden layers, where the upper and lower quartiles are determined by the PPTS(5) of the training and development set. The range between the upper and lower quartiles indicates the degree of bias and variance tradeoff; the smaller the range, the better the tradeoff. From Fig. 8b, one can find that the structure 20–15–1 has the best bias and variance tradeoff. Therefore, the model structure 20–15–1 was selected as the optimal model to predict IMF_1_.

**Figure 9:**
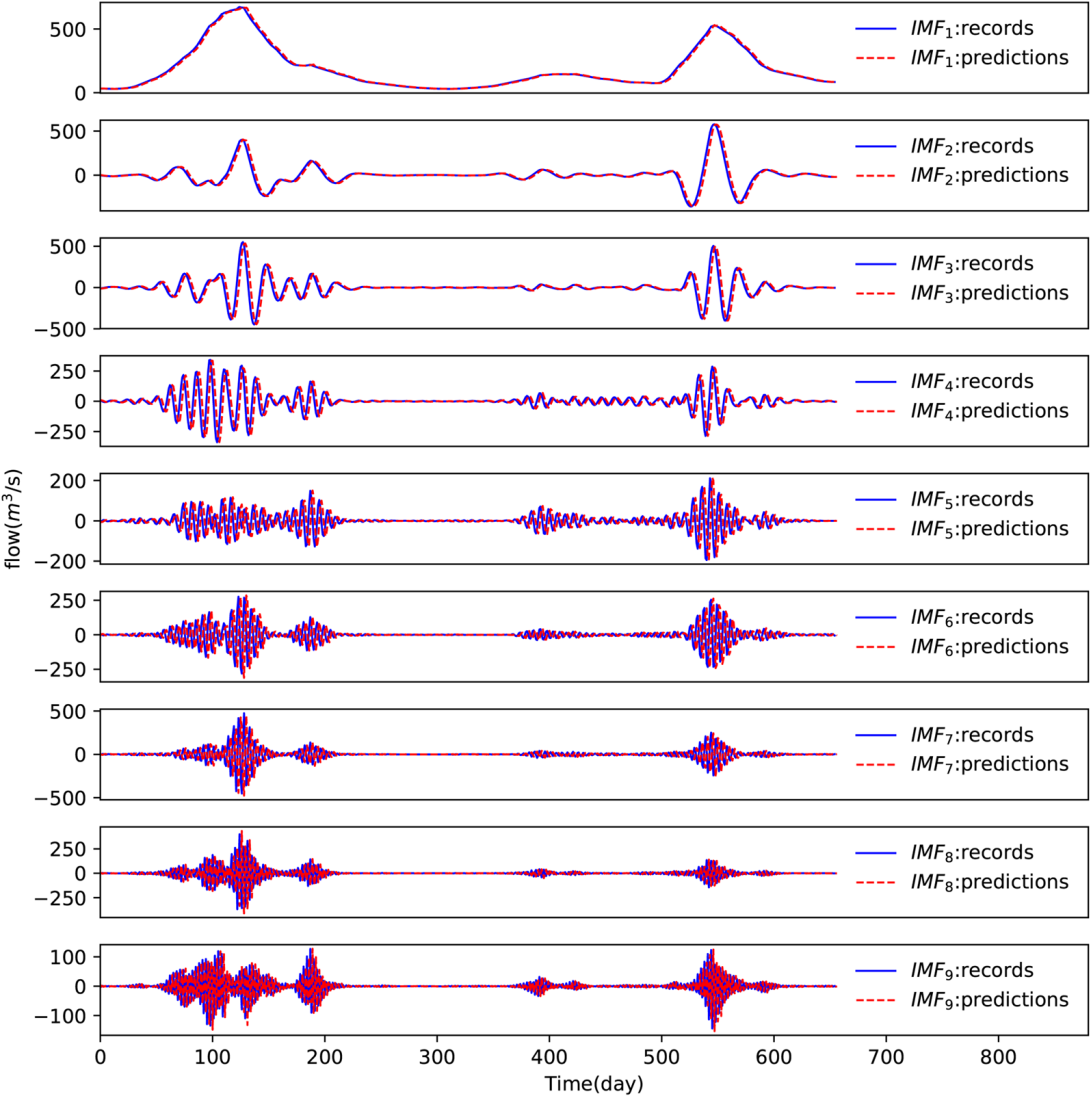
Forecasting result of sub-series during the testing period.

To validate the optimal model for forecasting each sub-series, the predictions during the test period were renormalized to the original scale and are plotted in Fig. 9. The PPTS(5) and R^2^ of the whole components during the training and development period are listed in Table 2. From Fig. 9 and Table 2, we can see that all the trained LSTM structures of all the sub-series have good accuracy.

**Table 2:** Results of evaluation criteria with different hidden layers and hidden units for sub-sequences.

Sequence	Hidden layers	Hidden units	Training		Developing	
			PPTS(5) (%)	R^2^	PPTS(5) (%)	R^2^
IMF1	1	15	0.0025	1.0000	0.0024	0.9999
IMF2	2	22	0.0396	0.9999	0.0227	0.9999
IMF3	1	16	0.2684	0.9999	0.2163	0.9997
IMF4	3	20	0.7646	0.9997	0.7156	0.9966
IMF5	4	20	0.8728	0.9993	0.2523	0.9983
IMF6	2	21	0.5031	0.9986	1.9367	0.9960
IMF7	2	23	1.2368	0.9973	0.4885	0.9943
IMF8	5	20	1.8371	0.9952	4.4562	0.9832
IMF9	1	25	1.5380	0.9972	2.1116	0.9897

### Training ensemble tree model GBRT

We can simultaneously build an ensemble tree, the GBRT model, to represent the relationship between the original series and the sub-series decomposed by VMD; the GBRT algorithm can learn an ensemble function to reconstruct all the sub-series into a streamflow series. To find the optimal hyper-parameters, Bayesian optimization based on Gaussian processes was applied^41,42^. The entire data set for building the GBRT mode, consisting of nine sub-series as input and raw streamflow as the output target, was divided into two parts: the training–validating set (1/1/1997–14/3/2013) and the testing set (15/3/2013–31/12/2014). The training–validating set is used to training the GBRT model and select the optimal parameters, while the testing set is used to validate the prediction performance of the models and to show the confidence level. The famous machine learning toolkit scikit-learn^43^ was applied to train the GBRT model by use of the training–validating set. To improve the performance of GBRT, sixfold cross-validation was used. The optimal value of the hyper-parameters for GBRT, i.e., learning rate, maximum depth, maximum features, minimum sample split and minimum sample leaf, were 0.08, 25, 9, 9 and 10, respectively. The performance evaluation results of RMSE = 45.9766, R^2^ = 0.9825, MAE = 11.4565 and PPTS(5) = 0.0438% indicate that GBRT had a good precision for multiscale feature ensembles.

### Predicting results of VMD-LSTM-GBRT

**Figure 10:**
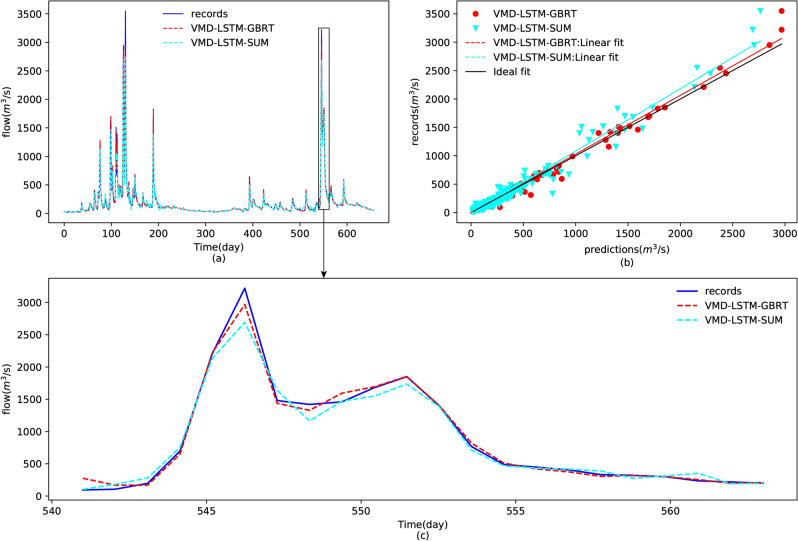
Comparison of prediction results of the test dataset using different ensemble techniques, GBRT and summation: (**a**) plots of the prediction results and records, (**b**) scatters for the test set (15/3/2013–31/12/2014), and (**c**) plots of the prediction results for the period 07/09/2014–28/09/2014.

Once the feature learning models based on LSTM were built, we could ensemble the sub-results to forecast the original streamflow. There are two ensemble methods: summation and GBRT. We first summed all nine sub-results obtained in “Multiscale deep feature learning with LSTM” to obtain the forecasting results of the original streamflow. The streamflows predicted by the model with ensemble summation, VMD-LSTM-SUM, are illustrated in Fig. 10. It can be observed from Fig. 10a that the predictions of a summation method can follow the changes of records during the test period but the accuracy of the peak streamflow forecasting is poor. Moreover, the scatter plot as shown in Fig. 10b indicates that the peak predictions are not appropriately concentrated near the ideal fit. The detailed plot of the predictions during the period 07/09/2014–28/09/2014 illustrated in Fig. 10c can also prove that point. By experiment, we found that the summation results of the nine components decomposed by VMD were quite different from the original streamflow at the peak. Therefore, we could not simply sum the predictions of the 9 components to forecast the original streamflow; the ensemble tree model obtained in “Training ensemble tree model GBRT”, GBRT, was applied to ensemble the sub-predictions predicted by LSTM. The final forecasting results forecast by the proposed model, VMD-LSTM-GBRT, are also shown in Fig. 10. From Fig. 10a, one can find that the peak predictions obtained by VMD-LSTM-GBRT are closer to the original streamflow. Moreover, the scatter plot as shown in Fig. 10b indicates that the predictions forecast by VMD-LSTM-GBRT concentrated near the ideal fit and agreed better with the records, which could also be proved by the small angle between the linear fit and the ideal fit. The predictions of the sub-set (07/09/2014–28/09/2014) of the test set shown in Fig. 10c denotes that the proposed model has better performance at the peak flow. Therefore, the proposed model, VMD-LSTM-GBRT, has a better capability of peak streamflow forecasting than VMD-LSTM-SUM.

To assess the forecasting performance of the proposed model, a different decomposition algorithm, EEMD, and two different feature learning models, DNN and SVR, were applied for the comparisons using the identical dataset shown in Fig. 4. The building process of these models is the same as the approach mentioned in “Multiscale deep feature learning with LSTM”, except that the decomposition algorithm and the feature learning model are replaced, respectively.

**Figure 11:**
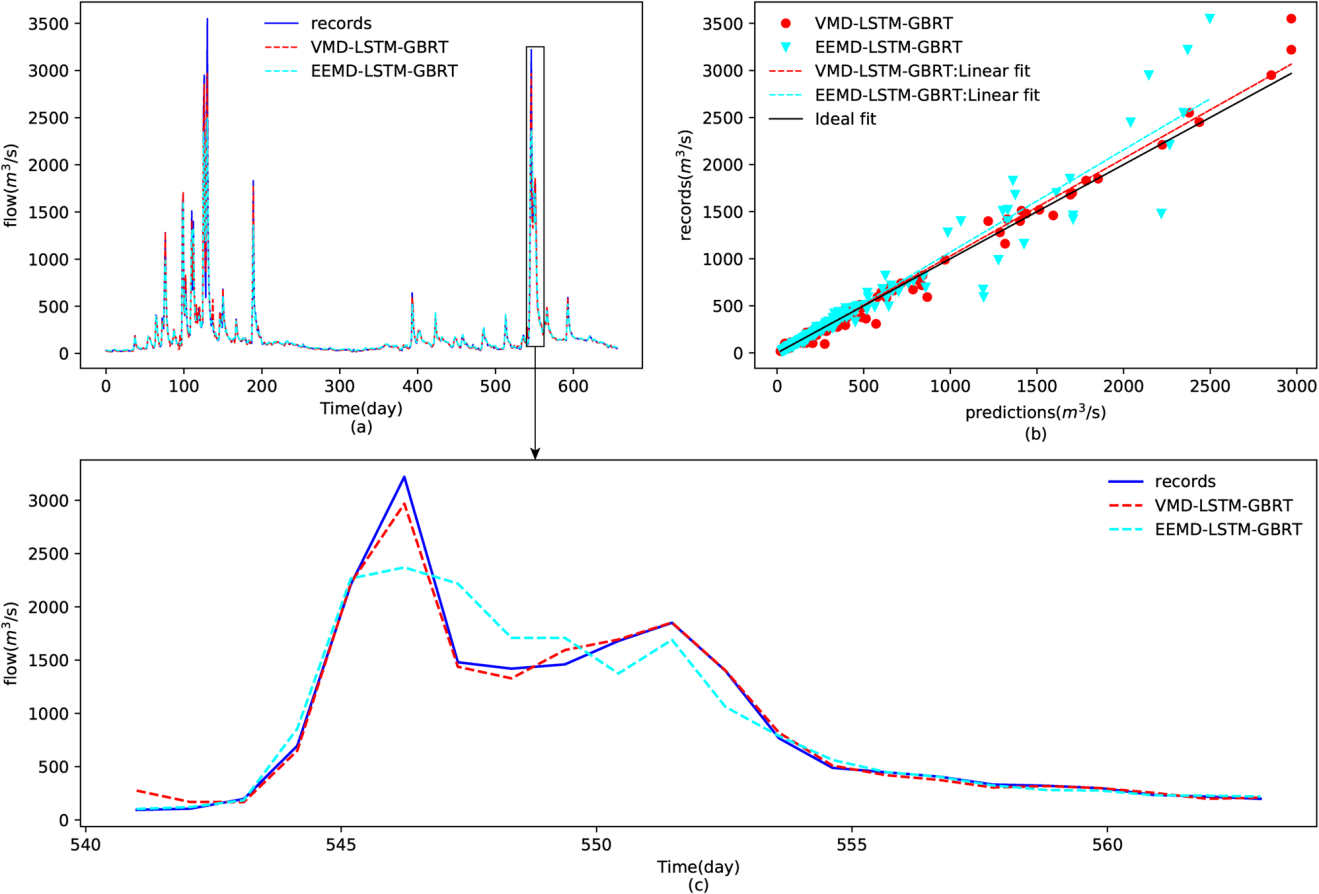
Comparison of prediction results of the test dataset using the different decomposition algorithms VMD and EEMD: (**a**) forecasting results, (**b**) scatters for the testing data (15/3/2013–31/12/2014), and (**c**) plots of the prediction results for the period 07/09/2014–28/09/2014.

**Figure 12:**
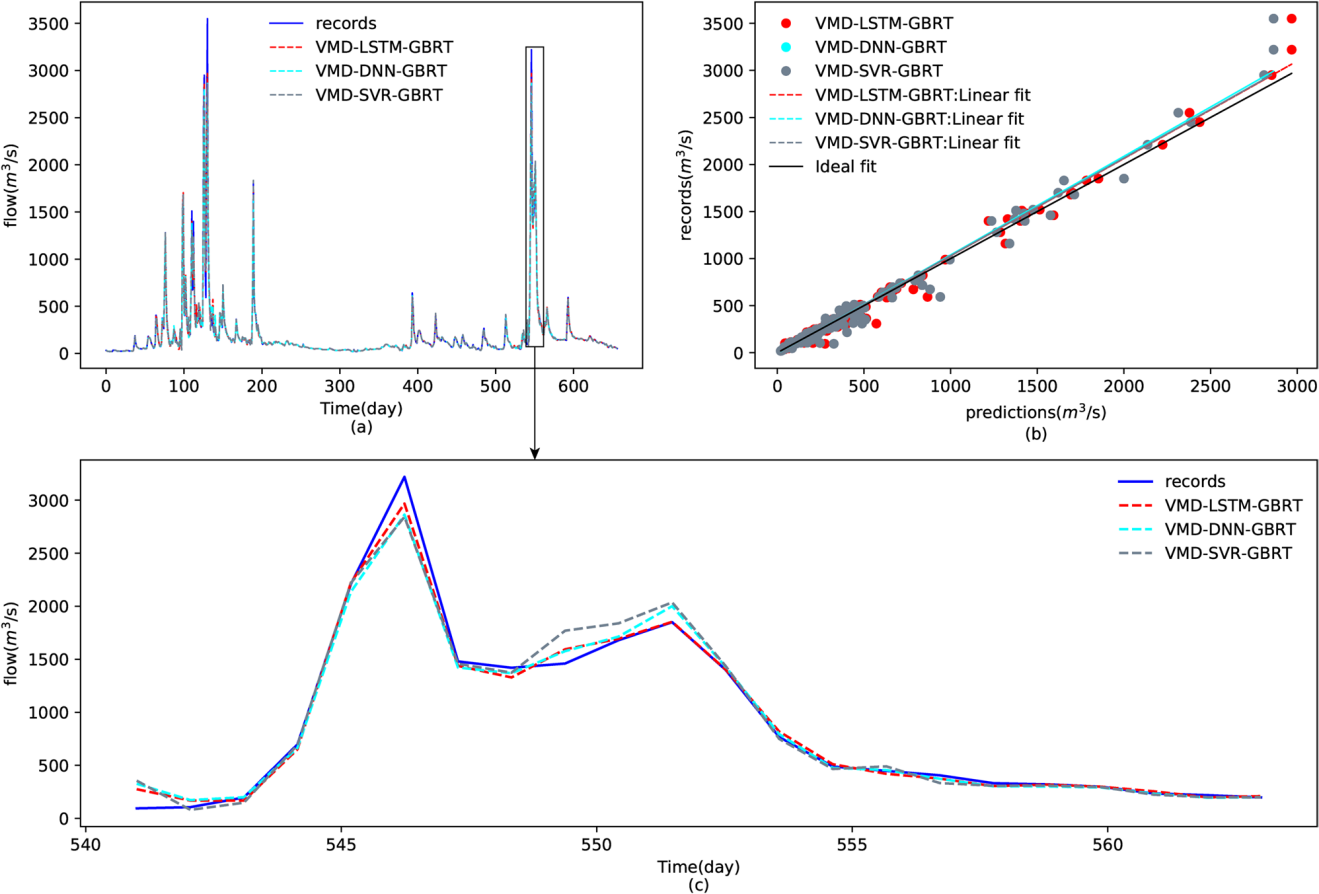
Comparison of forecasting results of the test dataset using the different feature learning models LSTM, DNN and SVR: (**a**) forecasting results, (**b**) scatters for the testing data (15/3/2013–31/12/2014), and (**c**) plots of the forecasting results during the period 07/09/2014–28/09/2014.

Figure 11 plots the streamflow predictions of Yangxian station by the proposed model VMD-LSTM-GBRT and the decomposition method-substituted model EEMD-LSTM-GBRT. As shown in Fig. 11a, the proposed model performed better for peak flow forecasting than the traditional decomposition method EEMD, which can be validated by Fig. 11c. From the scatter plot illustrated by Fig. 11b, one can find that the recorded predicted values of the proposed model are much more concentrated than the model using EEMD. Moreover, the comparison of prediction performance between the proposed feature learning model LSTM and the other two machine learning models, DNN and SVR, were conducted and are indicated by Fig. 12. However, from the forecasting results shown in Fig. 12a and the scatters shown in Fig. 12b, one can observe that the difference between the three feature learning models is not that obvious. However, we can still determine that the best feature learning model is the LSTM from the quantitative evaluations given in Table 3 and the detailed predictions shown in Fig. 12c. As shown in Table 3, the proposed model VMD-LSTM-GBRT has the lowest RMSE, MAE, PPTS(5) and the highest R^2^ among these decomposition-ensemble-based models, which illustrates the proposed model superiority for both peak streamflow forecasting and global changes.

**Table 3:** Comparison of the forecasting performances using different models.

Model	Performance criteria			
	RMSE	R^2^	MAE	PPTS(5) (%)
VMD-LSTM-GBRT	36.3692	0.9890	9.5246	0.0391
VMD-LSTM-SUM	67.8297	0.9619	27.8412	0.1500
EEMD-LSTM-GBRT	87.4506	0.9366	22.0321	0.0883
VMD-DNN-GBRT	44.9735	0.9832	12.1853	0.0451
VMD-SVR-GBRT	47.0555	0.9816	12.8919	0.0472
Linear regression	224.5310	0.5820	69.0201	0.2740
Multilayer perceptron	225.1806	0.5796	64.2869	0.1858

In the light of the above comparisons, all results fully indicate that the proposed model based on the decomposition algorithm, VMD, the multiscale deep feature learning model, LSTM, and the ensemble tree model, GBRT, performs very well for streamflow forecasting.

**Figure 13:**
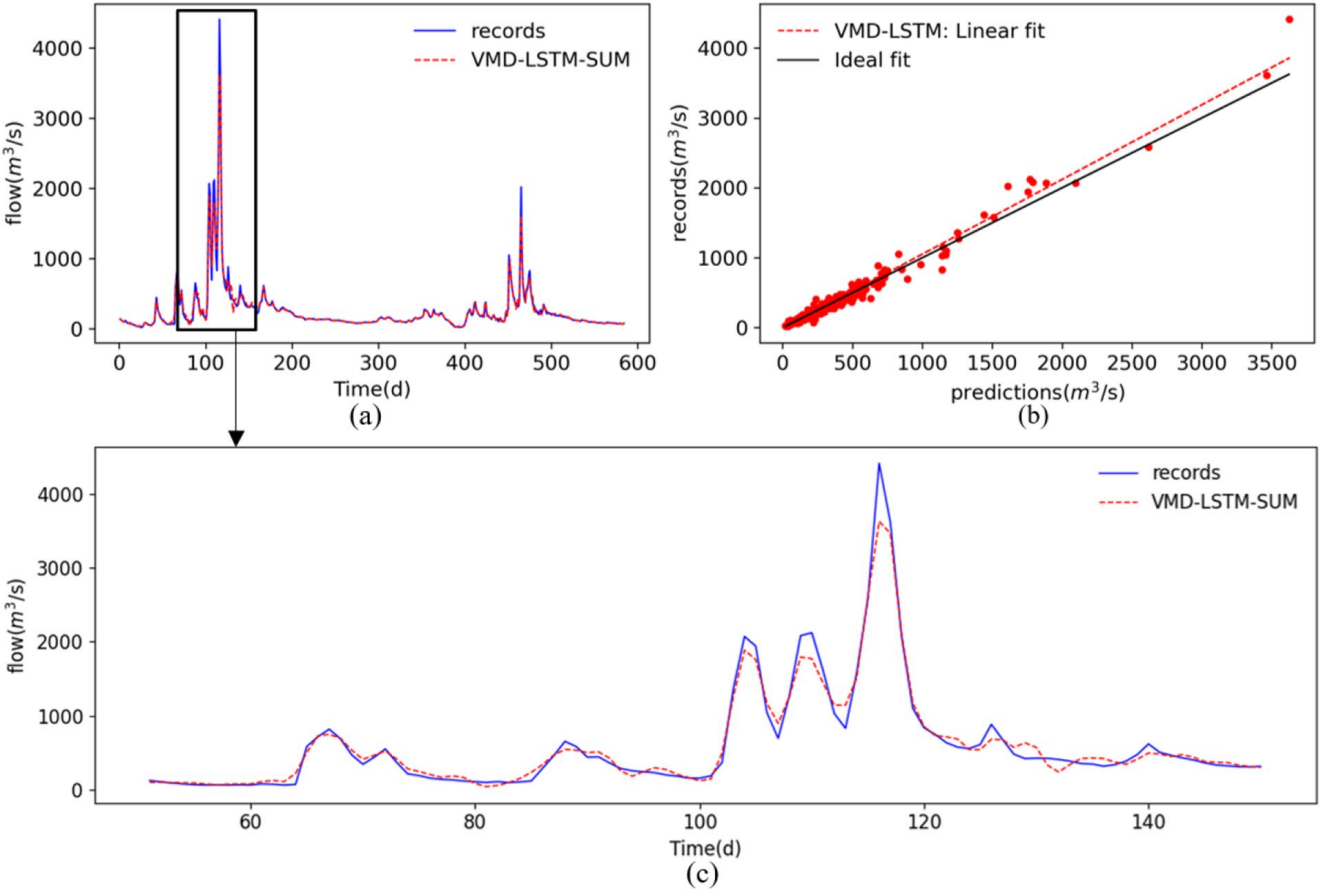
The forecasting results of the test dataset using VMD-LSTM-SUM: (**a**) forecasting results, (**b**) scatters for the testing data (15/3/2013–31/12/2014), and (**c**) plots of the forecasting results during the period 07/09/2014–28/09/2014.

In order to verify that the proposed method will obtain similar performance on other flow data sets, the model is applied to Huaxian hydrological station which is located at the Wei River Basin in China, the result is as shown in Fig. 13 and Table 4. The results show that the performance of the proposed method is consistent with that in Yangxian hydrological station.

**Table 4:** The forecasting performances using the proposed model VMD-LSTM-SUM on the Huaxian hydrological station.

Model	Performance criteria			
	RMSE	R^2^	MAE	PPTS(5) (%)
VMD-LSTM-SUM	57.5052	0.9754	22.5159	0.0725
Linear regression	148.0834	0.8363	43.1553	0.0951
Multilayer perceptron	141.5619	0.8505	40.7676	0.0931

## Conclusions

In this paper, a decomposition-ensemble-based multiscale feature learning approach with hybrid models was developed for forecasting daily streamflow, and the approach was evaluated based on a historical river streamflow dataset for Yangxian station, Han River, China. To improve the accuracy and stability of forecasting, three aspects were considered in a comprehensive way: (1) multiscale feature extraction by the algorithm with much more robustness with respect to sampling and noise; nine feature terms were extracted by VMD in this paper. (2) Deep feature learning with a model that can predict streamflow depending on the long historical changes of river flow; nine LSTMs were applied to sufficiently learn each feature. (3) An ensemble model with supervised learning; an ensemble tree GBRT was used to reconstruct the sub-results to obtain the final forecasting results. The daily streamflow forecasting capability of this decomposition-ensemble-based approach was compared with respect to three aspects: comparison with an approach that has the same feature learning model and ensemble technique but uses the traditional decomposition algorithm EEMD, comparison with an approach with the same decomposition algorithm and feature learning model but using the summation ensemble strategy, and comparison with an approach that has the same decomposition algorithm and ensemble technique but uses two different models, DNN and SVR. The results denote that the proposed model VMD-LSTM-GBRT exhibits the best forecasting performance among all the peer approaches for both global changes and peak streamflow forecasting.

This study proposes an approach to gain insight into the sophisticated features of natural river streamflow by designing a decomposition-ensemble framework. The three segments of this approach, i.e., using a robust model, VMD, to extract features that adequately represent natural river flow; using a long dependency model, LSTM, to remember or forget the historical changes of river flow; and reconstructing the extracted features by a tree model to remove the effect of error accumulation, are combined to determine what the values of the future streamflow should be. Note that the three segments of this framework can be replaced by other models, e.g., VMD can be replaced by an algorithm that is much more robust to sampling and noise but that still uses a regression strategy and replaces GBRT with DNN. Therefore, the present approach has value for river flow forecasting.

Streamflow forecasting is worthy of in-depth study. In the future, we will continue to study streamflow forecasting models. For instance, we could apply dynamic selection approaches^44,45^ to improve the ensemble’s performance in streamflow forecasting, and residual series modeling could be used to improve the accuracy of statistical and machine learning models^46,47^. It can make streamflow forecasting more and more accurate.
